# Quinine in Whooping Cough

**Published:** 1881-04

**Authors:** 


QUININE IN WHOOPING COUGH.—Dr. J. A. Mayes, of Mayesville, S. C., writes to Tilden's *Jounal of Materia Medica*, that he has "found nothing else to remove whooping cough so certainly and so quickly as quinine in small and repeated doses. In cases of very young infants one-sixth to one-fourth grain once in three hours. The cure is usually effected in a week or ten days.

